# Ribosomal S6 kinase (RSK) plays a critical role in DNA damage response via the phosphorylation of histone lysine demethylase KDM4B

**DOI:** 10.1186/s13058-024-01901-x

**Published:** 2024-10-21

**Authors:** Wenwen Wu, Jing Zhu, Naoe Taira Nihira, Yukiko Togashi, Atsushi Goda, Junki Koike, Kiyoshi Yamaguchi, Yoichi Furukawa, Takuya Tomita, Yasushi Saeki, Yoshikazu Johmura, Makoto Nakanishi, Yasuo Miyoshi, Tomohiko Ohta

**Affiliations:** 1 Department of Translational Oncology, St. Marianna University Graduate School of Medicine, 2-16-1, Sugao, Miyamae-ku, Kawasaki, 216-8511 Japan; 2 Department of Pathology, St. Marianna University School of Medicine, Kawasaki, Japan; 3 Division of Clinical Genome Research, The University of Tokyo, Tokyo, Japan; 4 Division of Protein Metabolism, The University of Tokyo, Tokyo, Japan; 5 Division of Cancer Cell Biology, The Institute of Medical Science, The University of Tokyo, Tokyo, Japan; 6 Division of Cancer and Senescence Biology, Cancer Research Institute, Institute for Frontier Science Initiative, Kanazawa University, Kanazawa, Japan; 7 Department of Surgery, Division of Breast and Endocrine Surgery, School of Medicine, Hyogo Medical University, Nishinomiya City, Hyogo Japan; 8 Department of Breast Medicine, Foshan Maternity & Child Healthcare Hospital, Southern Medical University, Foshan, China

**Keywords:** Ribosomal S6 kinase, RSK, KDM4B, DNA damage, Ionizing radiation, Breast cancer, Phosphorylation

## Abstract

**Background:**

Epigenetic dysregulation affecting oncogenic transcription and DNA damage response is a hallmark of cancer. The histone demethylase KDM4B, a factor regulating these processes, plays important roles in estrogen receptor-mediated transcription and DNA repair in breast cancer. However, how oncogenic phospho-signal transduction affects epigenetic regulation is not fully understood. Here we found that KDM4B phosphorylation by ribosomal S6 kinase (RSK), a downstream effector of the Ras/MAPK pathway, is critical for the function of KDM4B in response to DNA damage.

**Methods:**

KDM4B-knockout breast cancer cell lines were generated via CRISPR/Cas9-mediated gene editing. Re-expression of wild-type or phospho-site mutated KDM4B in knockout cells was performed by lentivirus-mediated gene transfer. Gene knockdown was achieved by RNA interference. DNA double-strand breaks (DSBs) were induced by ionizing radiation or laser-microirradiation. Protein accumulation at DSB sites was analyzed by immunofluorescence. KDM4B phosphorylation by RSK was assessed by in vitro and in vivo kinase assays. Gene and protein expression levels were analyzed by RT‒PCR and western blotting. The sensitivity of cells to ionizing radiation was examined by a clonogenic survival assay.

**Results:**

RSK phosphorylated KDM4B at Ser666, and inhibition of the phosphorylation by RSK depletion or RSK inhibitors abrogated KDM4B accumulation at the sites of DNA double-strand breaks (DSBs). DSB repair was significantly delayed in KDM4B-knockout cells or cells treated with RSK inhibitors. The replacement of endogenous KDM4B with the phosphomimetic mutant S666D restored KDM4B accumulation and DSB repair that had been inhibited by RSK inhibitors, suggesting a critical role for RSK at the specific serine residue of KDM4B in the effect of RSK inhibitors on DSB repair. As a consequence of these aberrant responses, inhibition of KDM4B phosphorylation increased the sensitivity of the cells to ionizing radiation.

**Conclusions:**

Overall, the present study uncovered a novel function of RSK on the DNA damage response, which provides an additional role of its inhibitor in cancer therapy.

**Supplementary Information:**

The online version contains supplementary material available at 10.1186/s13058-024-01901-x.

## Background

Epigenetic abnormalities affecting chromatin structure are a hallmark of cancer [1]. Chromatin compaction and relaxation mediated by histone modifications are crucial for the regulation of many oncogenic processes, including aberrant transcription and DNA damage response. Histone methylation, which is regulated by histone methyltransferases and histone lysine demethylases (KDMs), is the main histone modification that controls chromatin compaction [2]. Each KDM targets its specific histone residues, and KDM4 (also known as JMJD2) demethylates the most common ones, including Lys9 of histone H3 (H3K9) [3, 4]. The KDM4 family has four members, namely, KDM4A, KDM4B, KDM4C, and KDM4D, which may have some tissue specificity. For instance, KDM4B (JMJD2B) expression is significantly high in breast cancer compared with other cancers (Additional File 1: Fig. S1), suggesting its specific role in this cancer. KDM4B plays an oncogenic role in breast cancer by upregulating estrogen receptor α (ERα) signaling, whereas KDM4B is transcriptionally targeted by ERα, forming a feed-forward regulatory loop [5–7]. Previously, we found an essential role of KDM4B on ERα transactivation by mediating the interaction of ERα with steroid receptor coactivator (SRC) via its activation function-1 (AF-1) domain. KDM4B was degraded by a ubiquitin ligase SCF^Fbxo22^ in the presence of selective estrogen receptor modulators (SERM) in breast cancer endocrine therapy [8]. In addition to its role in transcription, KDM4B is critical in DNA damage response and affects the sensitivity of cells to chemotherapeutic agents [9–13]. Thus, KDM4B is recently considered a therapeutic target for breast cancer and other cancers [14].

Parallel to epigenetic dysregulation, cellular signal transduction pathways are often abnormal in cancer cells. Among them, aberrant stimulations of the Ras/MAPK and PI3K/AKT/mTOR pathways are major features of breast cancer [15]. Several kinases are involved in the pathways, and the p90 ribosomal S6 kinase (RSK) is thought to be a vital downstream effector of the Ras/MAPK pathway and is activated by ERK [16]. Significant cross-talks exist between the Ras/MAPK and PI3K/AKT/mTOR pathways via RSK [17, 18]. RSK phosphorylates and inhibits the tuberous sclerosis complex (TSC) as AKT does, promoting the activation of mTOR complex 1 (mTORC1) [19]. RSK also phosphorylates Raptor, an mTORC1 subunit, to stimulate the complex [20]. In addition, RSK directly phosphorylates ribosomal protein S6 (rpS6) and the translational initiation factor eIF4B, the most downstream targets of the PI3K/AKT/mTOR pathway, for translational initiation [21, 22]. Thus, RSK plays a pivotal role in the two major signal transduction pathways in breast cancer. However, how oncogenic phosphorylation signaling affects epigenetic regulation remains unclear.

Previous findings on the importance of KDM4B on ERα signaling in breast cancer prompted us to further investigate its role in other functions. In this study we found that phosphorylation of KDM4B by RSK was crucial for the function of KDM4B in DNA damage response. The inhibition of KDM4B phosphorylation including that mediated by RSK inhibitors abolished KDM4B accumulation at the sites of DNA double-strand breaks (DSBs), abrogated DSB repair, and resulted in increased sensitivity of cells to ionizing radiation. Our results revealed a novel role of RSK in DNA damage response and the potential application of its inhibition for cancer therapy.

## Methods

### Cell lines and culture conditions

Authenticated cell lines were obtained from the American Type Culture Collection and cultured according to the supplier’s instructions. MCF-7 and T-47D cells were further authenticated by short tandem repeat genotyping before genetic engineering. Cells were routinely monitored for mycoplasma infection using a Mycoplasma Detection Set (TaKaRa). Transfection of HEK293T cells was performed using standard calcium phosphate precipitation methods. For ionizing irradiation (IR), cells were exposed to X-ray irradiation at 2 Gy, unless otherwise described, and cultured for the indicated times before analysis. For cycloheximide (CHX)-chase assays, cells were treated with 50 μg/mL CHX for the indicated times.

### Chemicals

The chemical agents and their concentrations used in this study are listed in Additional File 1: Table S1. The concentrations of the agents were determined by preliminary examinations based on the manufacturer’s instructions.

### Plasmids

Complementary DNAs (cDNAs) of RSK1–RSK4 were cloned from a cDNA library of MCF-7 cells and inserted into a modified pcDNA3 vector containing an N-terminal Myc tag. KDM4B with N-terminal tandem-Strep-tag (St2-KDM4B) subcloned into pcDNA3 and HA-tagged constitutively active AKT1 with T308D and S473D mutations (HA-AKT1-2D) subcloned into pCS4 were described previously [23]. For lentivirus-mediated gene silencing or gene transfer, shRNAs were subcloned into CS-RfA-ETBsd, or St2-KDM4B was subcloned into CSIV-TRE-RfA-UbC-KT, respectively (both plasmids were generous gifts from Dr. Hiroyuki Miyoshi, RIKEN BioResource Center) [24]. S666A and S666D mutants were generated by site-directed mutagenesis.

### Knockout and stable expressions of KDM4B

For CRISPR/Cas9-mediated KDM4B knockout (KDM4B-KO), cells were transfected with pX458 comprising a human codon-optimized Cas9 (hSpCas9) nuclease along with a single-guide RNA (sgRNA, targeting sequence: 5’-CGTGGCCTACATAGAGTCGC-3’). CSIV-TRE-RfA-UbC-KT-puro was also co-transfected at 1/10 dose of pX458 for transient puromycin resistance. Cells were selected with 1 μg/mL puromycin for 3 days, and single colonies were picked up and further cultured. *KDM4B* mutation was confirmed by genetic sequencing and immunoblotting. To obtain a stable expression of wild-type (WT) or mutant St2-KDM4B in KDM4B-KO cells, cells were infected with lentivirus containing CSIV-TRE-RfA-UbC-KT-St2-KDM4B, which had been purified from HEK293T cells co-transfected with lentiviral vectors, and selected with 1 μg/mL puromycin. Cells were treated with 1 μg/mL doxycycline (Dox) for St2-KDM4B expression.

### Gene silencing by RNA interference

siRNA oligonucleotides targeting RSK1 (s12275), RSK4 (s26163) and a non-targeting control (4390847) were purchased from Thermo Fisher Scientific. RNA duplexes (10 nM) were transfected into cells using Lipofectamine RNAiMAX (Invitrogen) and analyzed 48 hours after transfection. To knock down Fbxo22 with shRNA, cells were infected with a lentivirus containing CS-RfA-ETBsd-shFbxo22 (5’-GGAATTGTAGTGACTCCAATG-3’), selected with 5 μg/mL blasticidin, and treated with 1 μg/mL Dox.

### Antibodies

The rabbit polyclonal antibody to the phosphorylated Ser666 residue of KDM4B (pS666-KDM4B) was generated against the phosphopeptide CKNRAApSFQAERKFN and purified by affinity purification and then absorption purification using an unphosphorylated peptide. The purchased antibodies used are listed in Additional File 1: Table S2.

### In vitro kinase assay

One microgram of recombinant full length KDM4B (Active motif) was co-incubated with 0.5 μg of recombinant active RSKs, AKT1 or ERK (SignalChem) in the presence or absence of 30 mM ATP at 30℃ for 10 min according to manufacture’s instruction. The reaction was terminated by boiling with the NUPAGE sample buffer and subjected to immunoblotting.

### Immunoprecipitation and immunoblotting

To analyze the steady-state levels of proteins in whole cell extracts, cells were lysed using radioimmunoprecipitation assay (RIPA) buffer, clarified, adjusted for protein concentration, boiled with the NUPAGE sample buffer, and subjected to immunoblotting. For the CHX-chase assays, the relative protein expression levels of KDM4B were measured using a densitometer and normalized to tubulin. For immunoprecipitation from the lysates containing the chromatin fraction, cells were incubated with 0.5% NP-40-based buffer [25] supplemented with 125 U/mL benzonase nuclease (Novagen) and 2 mM MgCl_2_ at 4 °C for 120 min, and the reaction was terminated with 5 mM EDTA, centrifuged, and the supernatants adjusted for protein concentration were used for immunoprecipitations, as described previously [26]. For the precipitation of St2-tagged proteins, StrepTactin^®^ resin (IBA-Lifesciences) was used as described previously [23, 26]. The precipitates were boiled with the NUPAGE sample buffer and subjected to immunoblotting.

### Laser microirradiation

To induce DSBs, cells were first sensitized with 10 μM bromodeoxyuridine (BrdU) for 12 h before irradiation. Laser microirradiation was performed using a PALM UV-A pulsed nitrogen laser (Carl Zeiss) as described previously [24, 27].

### Immunofluorescence microscopy

For indirect immunofluorescence, cells were pre-extracted using a buffer containing 20 mM HEPES (pH 7.5), 20 mM NaCl, 5 mM MgCl_2_, and 0.5% IGEPAL (A-630) supplemented with proteinase inhibitors and 200 μg/mL RNase A for 20 min on ice, washed with phosphate-buffered saline (PBS) solution, and fixed with 2% formalin in PBS for 20 min at room temperature. After blocking and incubation with primary and fluorescence-labeled secondary antibodies, the slides were mounted with ProLong Gold Antifade Mountant with DAPI (Invitrogen) and examined under a confocal laser scanning microscope (LSM 510 or 710, Carl Zeiss). The intensities of the proteins that accumulated at laser microirradiation-induced DSB sites were measured as described previously [27]. IR-induced γH2AX foci were mechanically counted using the Cellomics iDEV software (Thermo Fisher Scientific).

### RT-PCR

Total RNA from each sample isolated using an RNeasy kit (QIAGEN) was reverse-transcribed, and polymerase chain reaction was performed using the primers listed in Additional File 1: Table S3.

### Clonogenic survival assay

Cells were seeded at desired concentrations (5 × 10^2^ cells/well for HeLa and HCT116, 2 × 10^3^ cells/well for T47D and MCF7, and 1.5 × 10^4^ cells/well for ZR-751 and BT474) in 6-well plates and treated with the indicated IR dose. At 14–30 days after the IR, cells were fixed and stained with crystal violet. The colonies were scanned and counted using an ImageQuant LAS-4000 instrument (GE Healthcare). For the BI-D1870 treatment, the chemical was added 2 hours before IR, and cells were washed after 24 h and further cultured in the medium without the chemical.

### Statistical analyses

Statistical analyses were performed using a two-tailed Student’s *t*-test or two-way ANOVA using GraphPad Prism 8 (GraphPad Software Inc). *P* < 0.05 was considered significant.

## Results

### KDM4B accumulates at DSB sites and promotes DSB repair

**Fig. 1:**
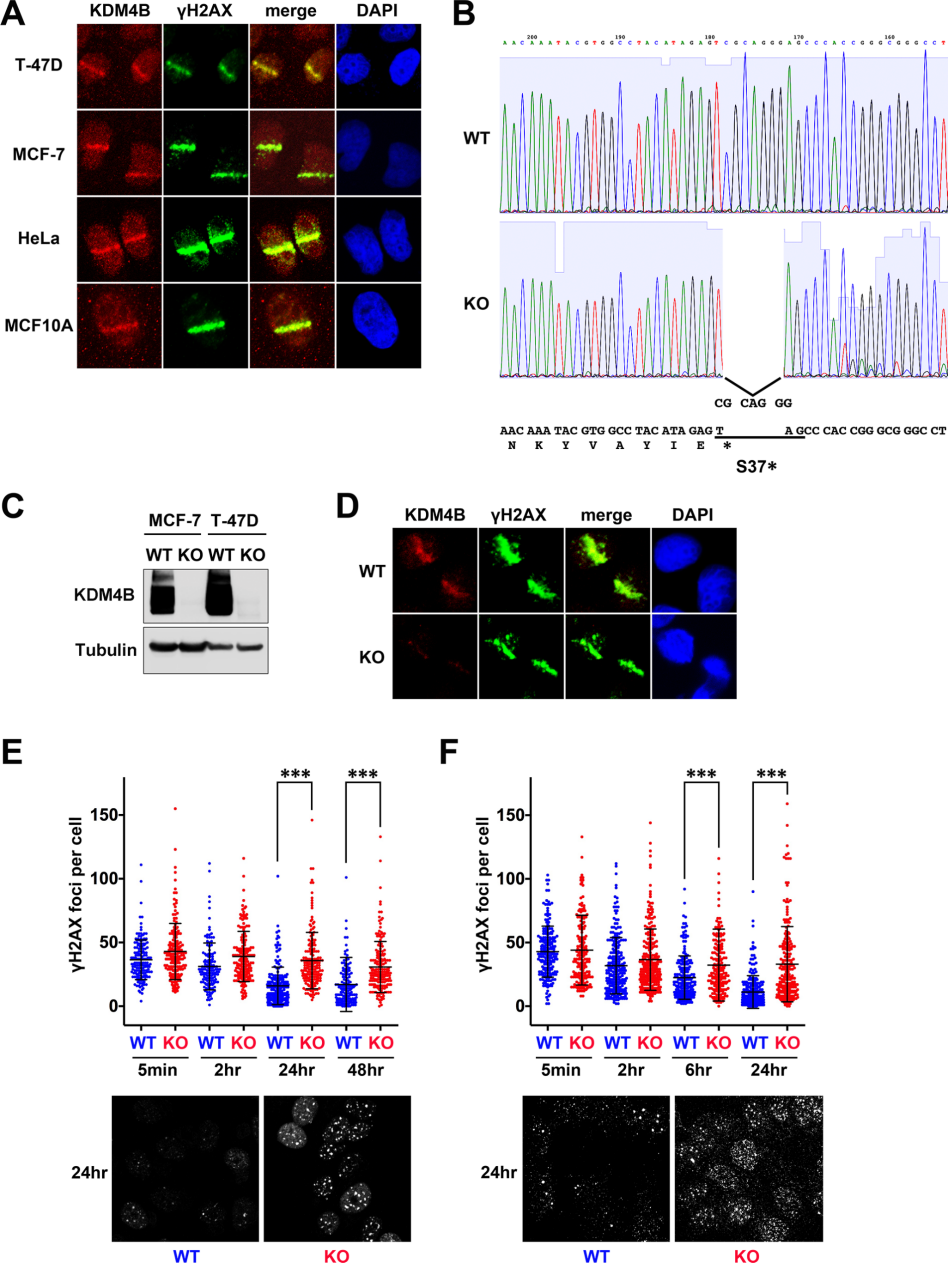
KDM4B accumulation at DSBs is crucial for DSB repair. (**A**) The indicated cells were treated with BrdU, laser microirradiated, and immunostained for KDM4B and γH2AX 30 min after microirradiation. Nuclei are counter-stained with DAPI. (**B**) CRISPR/Cas9-mediated knockout of KDM4B in T-47D cells. Sequencing chromatogram of cDNA shows the region with a deletion (CGCAGGG) that causes a premature stop codon at Ser37 in KDM4B-KO cells. (**C**) WT or KDM4B-KO MCF-7 and T-47D cells were subjected to immunoblotting with the indicated antibodies. (**D**) WT or KDM4B-KO T-47D cells were laser microirradiated and immunostained as in (**A**). (**E** and **F**) WT or KDM4B-KO T-47D (**E**) and MCF-7 cells (**F**) were irradiated and subjected to immunofluorescence analyses with the anti-γH2AX antibody at the indicated time after the IR. The number of nuclear γH2AX foci per cell is shown with means and SDs. ***P < 0.0001. Representative data 24 h after IR were shown at the bottom.

Previously it has been shown that exogenous KDM4B accumulated at DSB sites and was required for proper DNA damage response [9]. Therefore, whether endogenous KDM4B accumulates at the DSB sites was first verified. BrdU-treated cells were laser microirradiated, fixed after 30 min, and immunostained with antibody to KDM4B. In all the cell lines tested, KDM4B accumulated at the microirradiated sites co-localizing with a DSB marker γH2AX (Fig. 1A). To further analyze KDM4B functions, we next generated KDM4B-KO cells using CRISPR/Cas9 nuclease-mediated genome editing. Biallelic mutations causing immature stop codon(s) at the most N-terminal residues of KDM4B were created in two breast cancer cell lines: T-47D (Fig. 1B) and MCF-7 (Additional File 1: Fig. S2), and immunoblotting confirmed the deletion of the expression (Fig. 1C). The specificity of the anti-KDM4B antibody on the immunofluorescence of the microirradiated cells was confirmed using the knockout cell lines (Fig. 1D). Then, the requirement of KDM4B on DSB repair was examined. WT or KDM4B-KO T-47D and MCF-7 cells were irradiated, and after the IR, the number of IR-induced γH2AX foci was counted to evaluate recovery from the DSBs. Although the foci steadily declined in WT cells, they remained at significantly higher levels in KDM4B-KO cells for both T-47D and MCF-7 cells (Fig. 1E and F). These results indicate that KDM4B accumulates at the DSB sites and plays an important role in DSB repair.

### RSK phosphorylates and stabilizes KDM4B

**Fig. 2:**
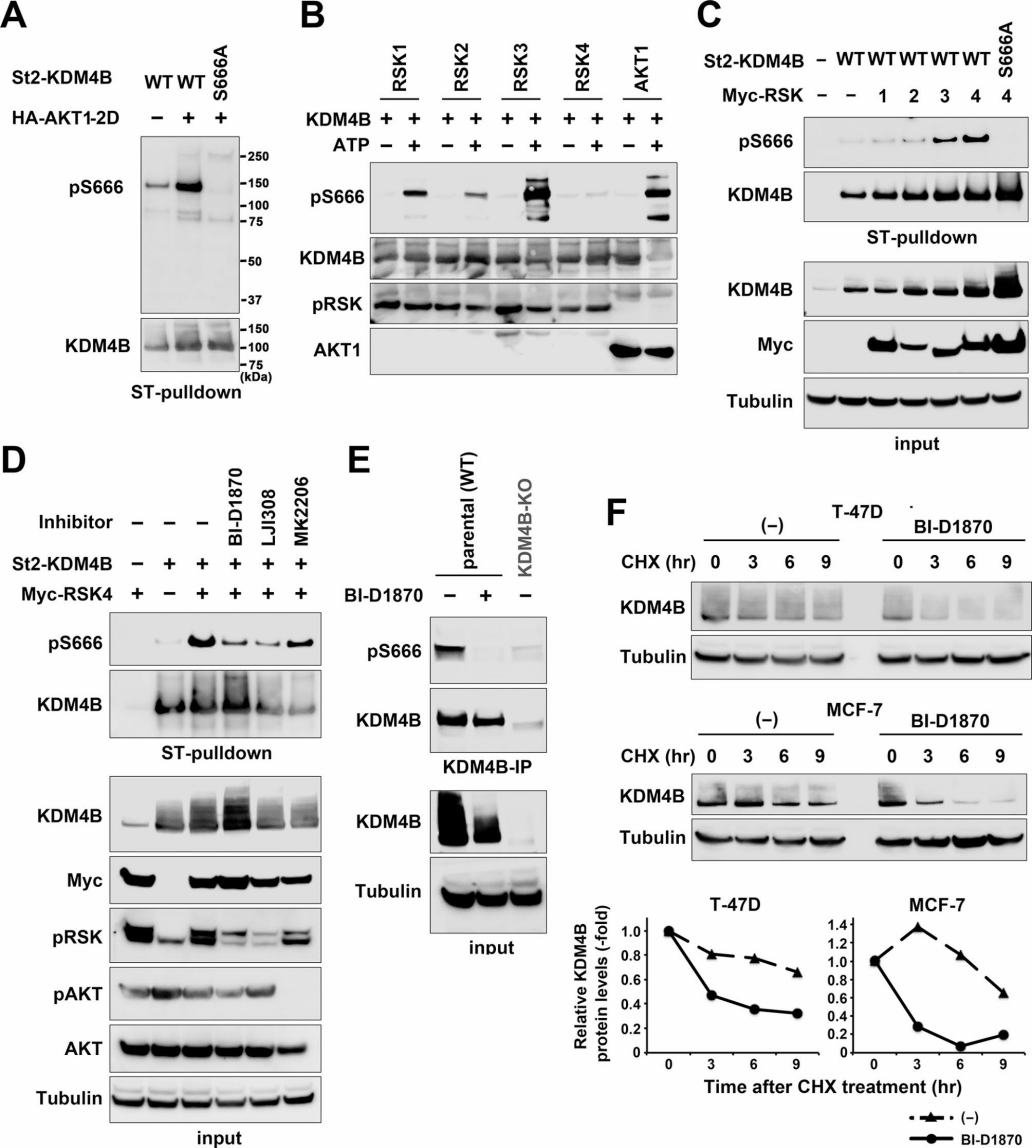
Effect of RSKs on KDM4B phosphorylation and stability. (**A**) HEK-293T cells were transfected with WT or S666A mutant of St2-KDM4B with or without HA-AKT1-2D and subjected to StrepTactin (ST) pulldown, followed by immunoblotting with the indicated antibodies. (**B**) The purified KDM4B protein was incubated with RSKs or AKT1 with or without ATP. The reaction was then subjected to immunoblotting with the indicated antibodies. pRSK, pan phospho-RSK. (**C** and **D**) In vivo phosphorylation of KDM4B at Ser666 was detected as in (**A**) with Myc-RSK1 to Myc-RSK4 (**C**) or Myc-RSK4 and kinase inhibitors (**D**) as indicated. Inputs were also loaded. (**E**) WT or KDM4B-KO T-47D cells were treated with or without BI-D1870 for 24 h and subjected to immunoprecipitation with anti-KDM4B antibody, followed by immunoblotting. (**F**) T-47D and MCF-7 cells treated with or without BI-D1870 for 24 h were incubated with CHX for the indicated times and subjected to direct immunoblotting. Relative KDM4B expression levels are shown in the lower panels.

Previously, we found that AKT phosphorylates KDM4B at the Ser666 residue, and the phosphorylation protects KDM4B from Fbxo22-mediated ubiquitination and degradation [23]. To further investigate the significance of the phosphorylation, we established a rabbit polyclonal antibody against pS666-KDM4B. To verify the specificity of the antibody for immunoblotting, St2-KDM4B was expressed ectopically in HEK-293T cells, and the protein was pulled down with StrepTactin and immunoblotted with the antibody. The antibody clearly detected bands that were enhanced by co-transfection of a constitutively active AKT1 mutant and eliminated by the substitution of the WT St2-KDM4B with a non-phosphorylatable S666A mutant (Fig. 2A). Because the residues around S666 (KNRAApSF) matches not only with a consensus motif for the phosphorylation site of AKT but also that of RSKs [17], whether RSKs can phosphorylate KDM4B was tested. The RSK family consists of four closely homologous members, RSK1–RSK4, which promote overlapping but distinct functions through differential expression in tissues [16]. Purified recombinant full-length KDM4B was incubated with recombinant RSKs, AKT1, and ERK proteins with or without ATP, and the reactions were subjected to immunoblotting with the anti-pS666-KDM4B antibody. RSKs and AKT1 phosphorylated KDM4B in the in vitro reaction, in which RSK3 activity was at maximum and RSK4 activity was very low (Fig. 2B and Additional File 1: Fig. S3A). Interestingly, however, in vivo, RSK4 exhibited the highest activity on phosphorylation, whereas other RSKs possess limited activity (Fig. 2C). The reason for the discrepancy is currently unknown; however, a potential reason was that, RSKs essentially require PDK1 and ERK for their activation, but unlike other RSKs, RSK4 does not require endogenous PDK1 for its activity, and a low ERK activity level is sufficient [28], whereas the purchased purified RSKs were pre-activated. Although RSKs are downstream of the EGFR/MAPK/ERK pathway, the addition of epidermal growth factor (EGF) did not enhance the activity of RSKs on pS666-KDM4B (Additional File 1: Fig. S3B and S3C). We next tested the effect of RSK and AKT inhibitors on RSK4-induced KDM4B phosphorylation. The activity and specificity of the inhibitors were verified with their effects on the autophosphorylation of RSK and AKT (Additional File 1: Fig. S3D). Although the RSK inhibitors BI-D1870 and LJI308 remarkably reduced KDM4B phosphorylation, the AKT inhibitor MK2206 showed only limited effects (Fig. 2D and Additional File 1: S3C). The effect of BI-D1870 on endogenous pS666-KDM4B was also verified. Phosphorylation was detected in WT cells without any stimulation, but not in KDM4B-KO cells, and was inhibited by BI-D1870 (Fig. 2E). During the analyses using BI-D1870, we noticed that steady state level of endogenous KDM4B was reduced by the treatment (Fig. 2E, input). This finding suggests that RSKs may stabilize KDM4B by phosphorylation-mediated protection from degradation by Fbxo22 as AKT did [23]. Indeed in the CHX-chase assays, the protein half-lives of KDM4B were significantly reduced by BI-D1870 in MCF-7 and T-47D cells, indicating that RSK inhibition decreases KDM4B stability (Fig. 2F). These results suggest that KDM4B is physiologically phosphorylated by RSKs, and phosphorylation is critical for KDM4B stability.

### RSK is critical for KDM4B accumulation at DSB sites

**Fig. 3:**
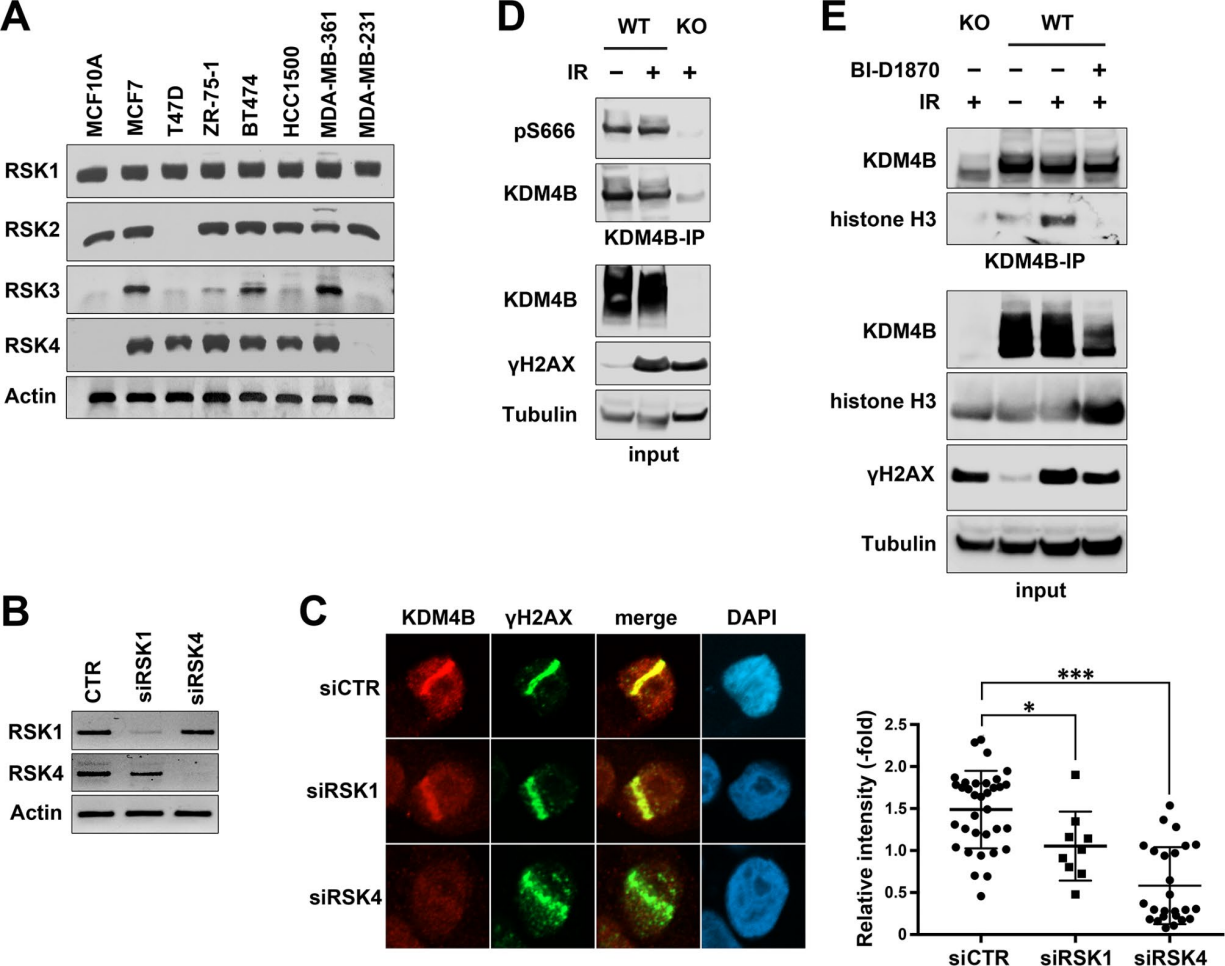
Effect of RSKs on KDM4B accumulation at DSBs. (**A**) The indicated cells were subjected to RT-PCR to determine the expression of RSKs. Actin was used as a loading control. (**B** and **C**) T-47D cells were transfected with either control (CTR) siRNA or that targeting RSK1 or RSK4 and subjected to RT-PCR (**B**) or laser microirradiation followed by immunofluorescence as in Fig. 1A (**C**). The relative intensities of KDM4B are shown as dot plots with means and SDs in the right panel. ***P < 0.0001, *P < 0.02. (**D** and **E**) WT or KDM4B-KO T-47D cells were treated with or without BI-D1870 and irradiated as indicated and after 30 min subjected to immunoprecipitation (IP) with anti-KDM4B antibody, followed by immunoblotting. Inputs were also loaded.

The DNA damage response of KDM4B and its phosphorylation by RSK led to the investigation of whether this phosphorylation influences KDM4B function in DNA damage response. To clarify the effect of RSKs on KDM4B function, the expression levels of RSK family members in breast cancer cell lines were initially analyzed. The expression varies among cell lines, whereas MCF-7 expressed all four members of RSK, T-47D expressed only RSK1 and RSK4 (Fig. 3A). T-47D cells were used for siRNA knockdown analyses to minimize the complementary effects of other RSK family members. RT-PCR confirmed the effectiveness of siRNAs on the depletion of RSKs (Fig. 3B). Cells were then laser microirradiated and subjected to immunofluorescence. KDM4B accumulation at γH2AX-labeled DSB sites were significantly reduced with the depletion of both RSK1 and RSK4; however, the effect of RSK4 depletion was greater than that of RSK1 depletion (Fig. 3C). It was not likely that the DNA damage induced phosphorylation because pS666-KDM4B was detectable without any treatment, and IR did not enhance the phosphorylation (Fig. 3D). However, IR treatment increased the interaction of KDM4B with a nucleosome component histone H3, and this interaction was blocked by BI-D1870 (Fig. 3E). These results suggest that the RSK-mediated Ser666 phosphorylation is a prerequisite for KDM4B accumulation at DSB sites.

### RSK inhibitors inhibit KDM4B accumulation and function at DSB sites

**Fig. 4:**
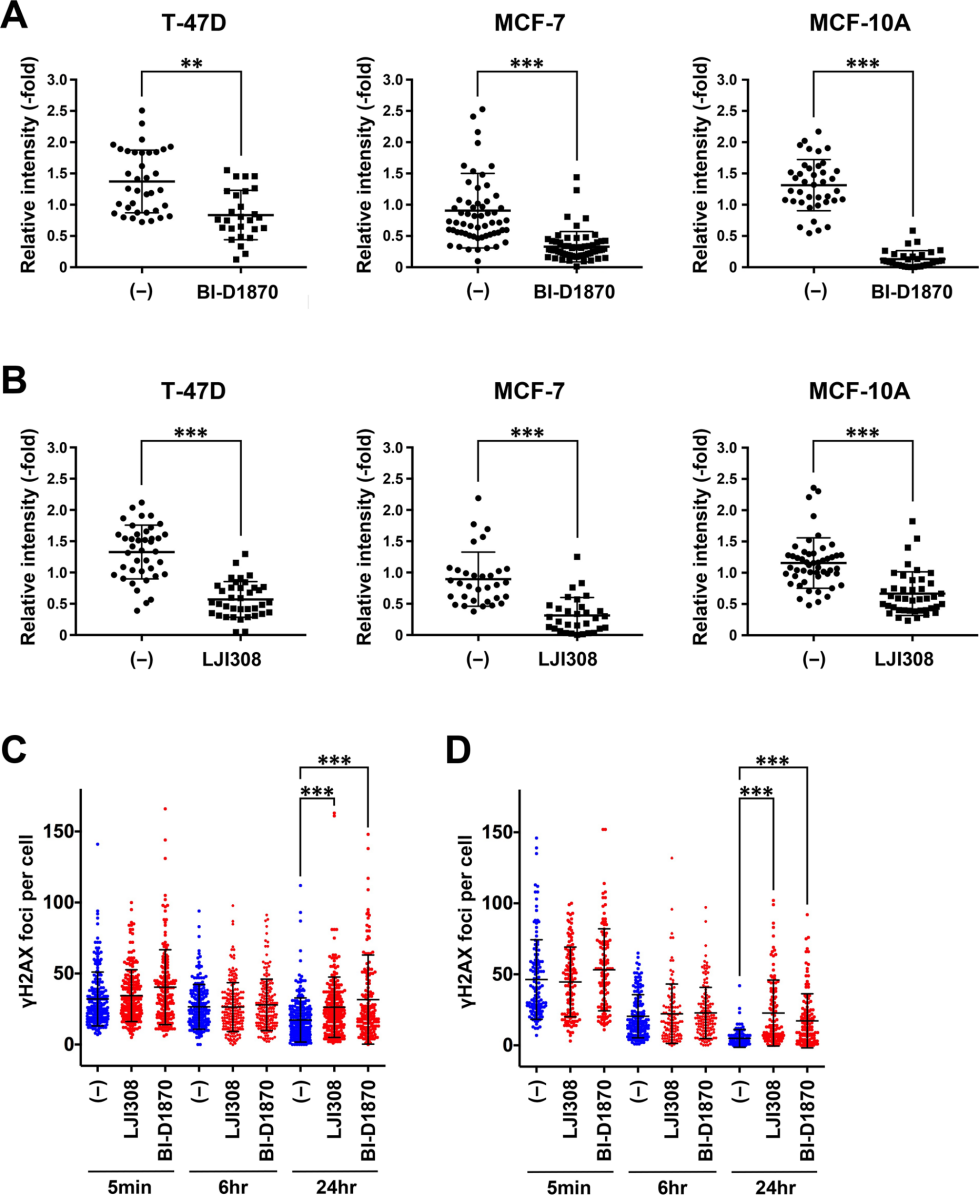
Effect of RSK inhibitors on KDM4B function in response to DNA damage. (**A** and **B**) The indicated cells were treated with or without BI-D1870 (**A**) or LJI308 (**B**) for 24 h, laser microirradiated, and subjected to immunofluorescence as in Fig. 1A at 30 min (**A**) or 2 h (**B**) after microirradiation. The relative intensities of KDM4B are shown with means and SDs. **P < 0.001, ***P < 0.0001. (**C** and **D**) T-47D (**C**) or MCF-7 cells (**D**) were incubated with the indicated RSK inhibitors and analyzed for recovery of IR-induced γH2AX foci as in Fig. 1E and F. ***P < 0.0001

We next analyzed the effect of RSK inhibitors on KDM4B accumulation and function at DSB sites. T-47D, MCF-7, and MCF-10 A cells were treated with or without the RSK inhibitor BI-D1870 or LJI308, incubated with BrdU and laser microirradiated (Fig. 4A and B, Additional File 1: S4A, and S4B). Consistent with the RSK siRNA experiments, KDM4B accumulation at DSB sites was significantly reduced by RSK inhibitors in all cell lines. BI-D1870 inhibited KDM4B retention as early as 30 min after laser microirradiation, whereas the effect of LJI308 on KDM4B retention at DSB sites emerged relatively later. In contrast, the AKT inhibitor MK2206 did not inhibit KDM4B accumulation (Additional File 1: Fig. S4C). Consequently, treatment with the RSK inhibitor BI-D1870 or LJI308 significantly delayed DSB recovery as analyzed by the γH2AX foci in the two cell lines (Fig. 4C and D). The results suggest that RSK inhibitors prevent KDM4B accumulation at DSB sites and compromise cells’ ability to repair DSBs, possibly through KDM4B dysfunction.

### RSK inhibitors compromise DSB repair via the inhibition of KDM4B phosphorylation

**Fig. 5:**
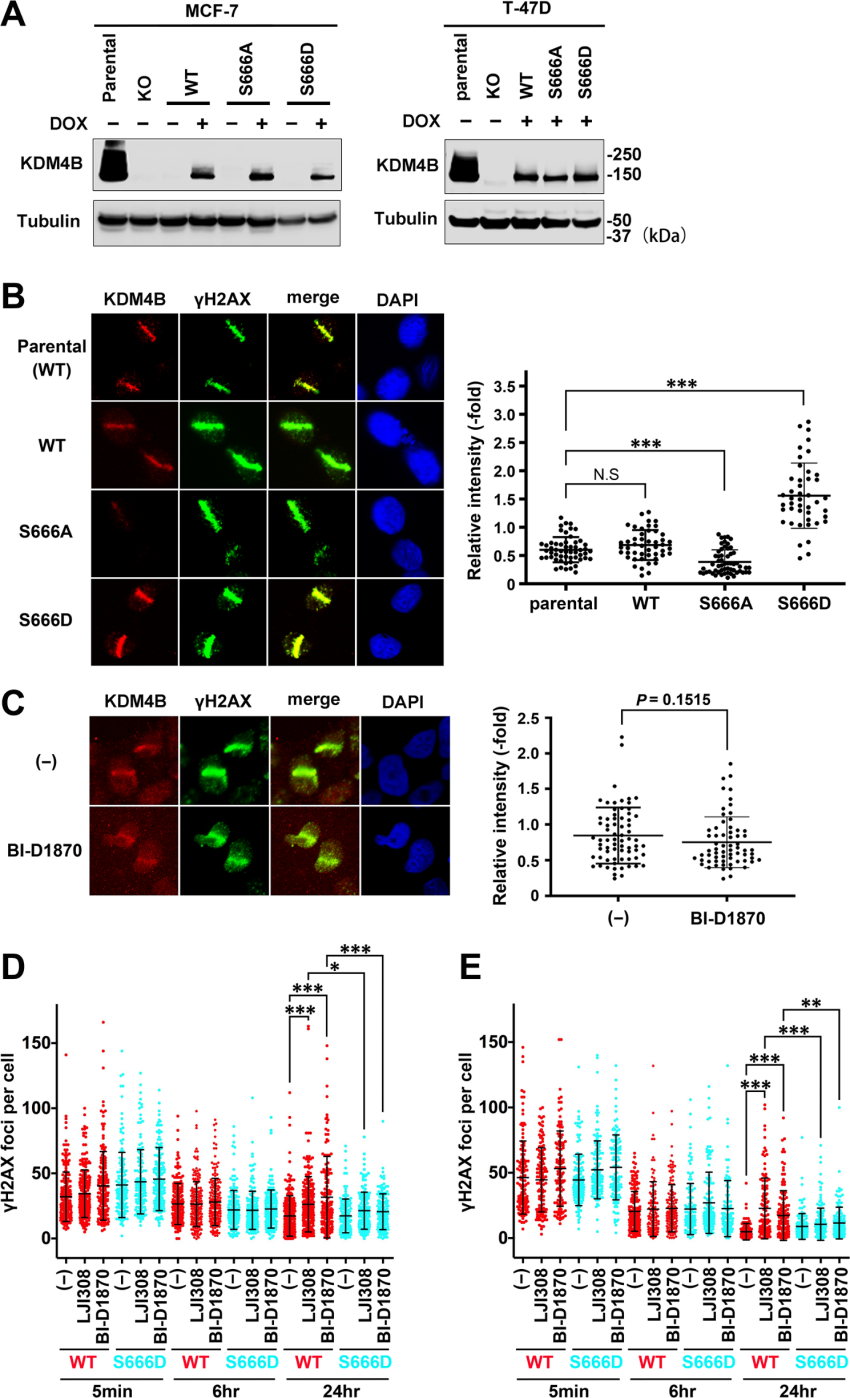
Effects of phospho-site mutations on KDM4B function in response to DNA damage. (**A**) Parental WT or KDM4B-KO MCF-7 and T-47D cells, or KDM4B-KO cells add-backed with WT or S666A/D mutant KDM4B, were cultured with or without Dox and subjected to immunoblotting with the indicated antibodies. (**B** and **C**) Parental T-47D or KDM4B-KO cells expressing exogenous KDM4B as indicated (**B**) or KDM4B-KO cells expressing S666D mutant incubated with or without BI-D1870 (**C**) were laser microirradiated followed by immunofluorescence analysis as in Fig. 1A. The relative intensities of KDM4B are shown with means and SDs in the right panels. ***P < 0.0001. N.S., not significant. (**D** and **E**) Either WT or KDM4B-S666D T-47D (**D**) and MCF-7 cells (E) were incubated with the indicated RSK inhibitors and analyzed for the recovery of IR-induced γH2AX foci as in Fig. 1E and F. *P < 0.01, **P < 0.002, ***P < 0.0001

To clarify whether Ser666 phosphorylation of KDM4B is responsible for the observed effect of RSK inhibitors on DSB repair, cell lines that constitutively express WT, non-phosphorylatable S666A, or phosphomimetic S666D mutant of KDM4B in a Dox-inducible manner were established from KDM4B-KO T-47D and MCF-7 cells (Fig. 5A). Supporting the phosphorylation-dependent accumulation of KDM4B, KDM4B accumulated at γH2AX-labeled DSB sites in S666D cells at significantly higher levels than in parental WT cells, whereas the accumulation was at similar or significantly lower levels in WT (add-back) or S666A cells, respectively (Fig. 5B). Although each exogenous KDM4B was expressed at similar levels (Fig. 5A), KDM4B accumulation at DSB sites was significantly reduced in S666A cells. This suggests that destabilization is not the only reason why unphosphorylated KDM4B does not accumulate at DSB sites. To verify this point, we depleted Fbxo22 with shRNA to stabilize KDM4B. Fbxo22 depletion increased the steady-state level of KDM4B-S666A (Additional File 1: Fig. S5A) but did not allow KDM4B-S666A to accumulate at DSB sites (Additional File 1: Fig. S5B), indicating that KDM4B phosphorylation is critical for its retention at DSB sites by some mechanisms in addition to stabilization. Notably, the RSK inhibitor BI-D1870 did not reduce KDM4B-S666D accumulation at DSB sites (Fig. 5C), indicating that the reduction of KDM4B retention at DSB sites by the inhibitor (Fig. 4A) was through inhibition of Ser666 phosphorylation. Next the effect of RSK inhibitors on DSB repair was tested using this phosphomimetic S666D cell lines. Parental WT or KDM4B-S666D cells were incubated with or without RSK inhibitors and irradiated, and γH2AX foci were counted after IR. Although the foci steadily declined in untreated WT cells, they remained at significantly higher levels in those treated with BI-D1870 or LJI308 for both MCF-7 (Fig. 5D) and T-47D (Fig. 5E) cells. Importantly, however, this effect of BI-D1870 or LJI308 was canceled in S666D cells. This finding indicates that the inhibition of DSB repair by RSK inhibitors is mediated by the inhibition of KDM4B phosphorylation. Together, these results suggest that RSK is crucial in the DNA damage response via KDM4B phosphorylation and that the RSK inhibitor can interfere in this process.

### Inhibition of RSK-mediated KDM4B phosphorylation sensitizes cells to IR

**Fig. 6:**
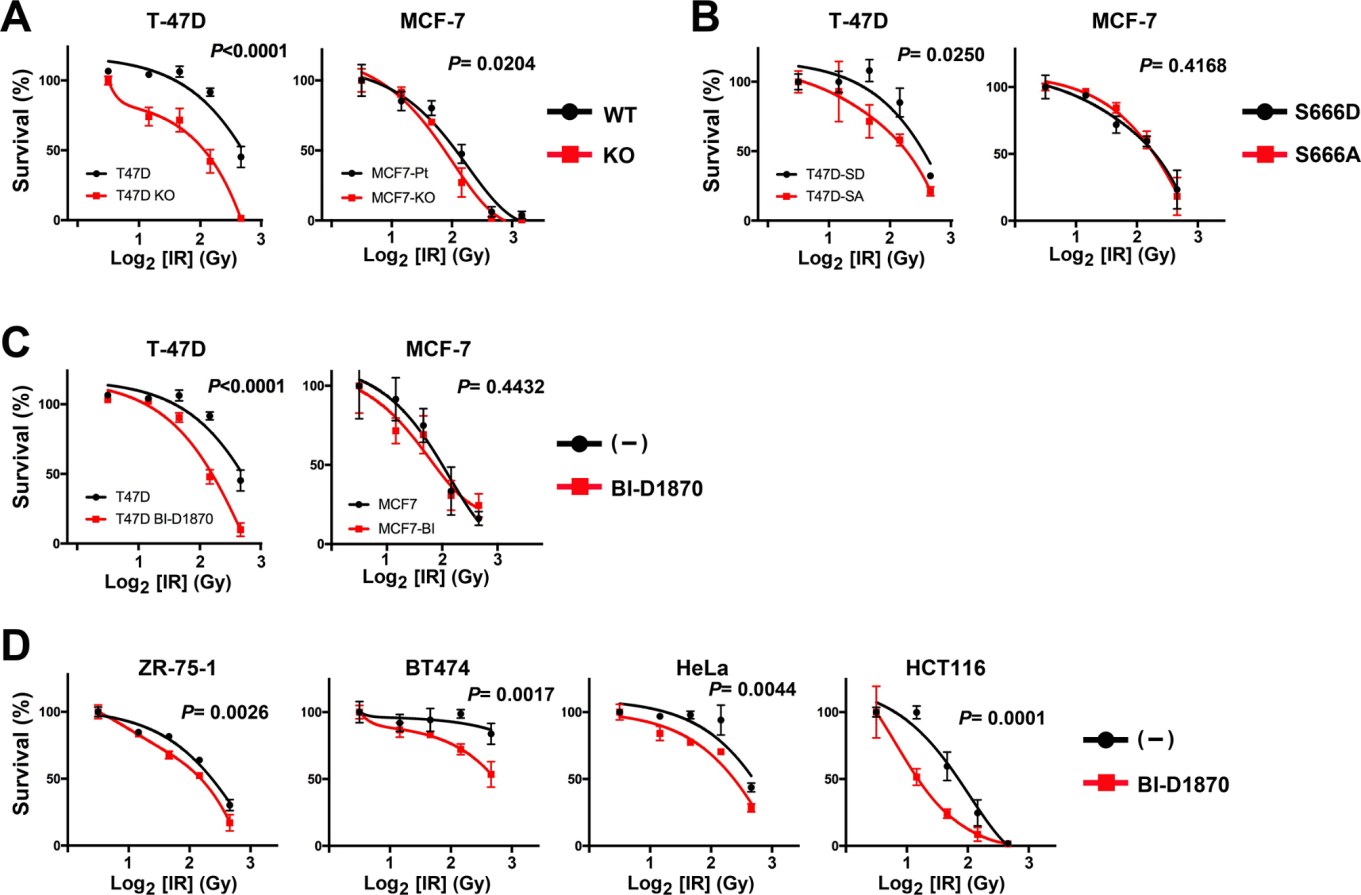
Sensitivity of cells with different KDM4B status to IR. The indicated cells were irradiated at different doses (0, 1.25, 2.5, 5, and 10 Gy) and analyzed for clonogenic survival after 2 (for HeLa and HCT116) or 3 (for others) weeks. (**A**) WT vs. KDM4B-KO. (**B**) KDM4B-S666D vs. S666A. (**C** and **D**) WT cells with vs. without BI-D1870. Relative survivals from triplicate experiments are shown as averages ± SD. Statistical significances were calculated using the two-way ANOVA.

Because the inhibition of RSK-mediated KDM4B phosphorylation delayed the repair of IR-induced DSBs, we analyzed whether it affects cell sensitivities to IR. First, the effect of KDM4B deletion was confirmed. WT or KDM4B-KO T-47D and MCF-7 cells were exposed to a range of IR doses and allowed to form colonies. KDM4B depletion significantly sensitized the cells to IR (Fig. 6A). Then, the sensitivities of cells with S666A and S666D mutants of KDM4B were compared. S666A-mutant cells were significantly sensitive to IR compared with the S666D cells in T-47D cells (Fig. 6B). BI-D1870 also increased the sensitivity of T-47D cells, although it was not significant for MCF-7 cells (Fig. 6C). Because these effects on MCF-7 cells were somewhat limited, other cancer cell lines were further examined to verify the effect of BI-D1870 on IR sensitivity. As shown in Fig. 6D, BI-D1870 significantly increased the sensitivity of all cell lines tested including ZR-75-1 and BT474 breast cancer cells, as well as HeLa and HCT116 cells, to IR. These data suggest that the inhibition of KDM4B phosphorylation by RSK results in the aberrant repair of IR-induced DNA damage and therefore increases the sensitivity of cells to IR.

## Discussion

In the present work, we showed that KDM4B is phosphorylated by RSK, and the modification is critical for its protein stability, retention in chromatin with DSBs and repair of the breaks. DNA damage did not induce this phosphorylation, and prior stabilization of KDM4B by phosphorylation may be a requirement for KDM4B accumulation at DSB sites. However, stabilization is not the only role of phosphorylation because non-phosphorylatable S666A mutants did not accumulate at DSB sites even after their steady-state levels were increased by Fbxo22 depletion. The detailed mechanism for the phosphorylation-dependent retention still requires clarification.

Although AKT1 phosphorylates the same Ser666 residue of KDM4B as RSK does, only RSK inhibition, but not AKT1 inhibition, could prevent KDM4B retention at DSB sites. It has been reported that KDM4B demethylates and inactivates AKT1 that was activated by SETDB1-mediated methylation [29, 30]. To this end, KDM4B interacts with AKT1 through its Tudor domain, which may competitively prevent KDM4B from interacting with chromatin via the methylated histone H4K20me2. Another possible reason is that although AKT1 is activated after IR [31], this induction of AKT1 activity may not be fast enough for KDM4B recruitment, and RSK may have higher basal activities to KDM4B than AKT1. In this regard, RSK4 differs from other RSKs because its constitutive activity does not require signal-induced ERK activity or PDK1 [28, 32]. Because BI-D1870 possesses stronger inhibitory activity against RSK4 than against other RSKs [33], it is possible that RSK4 mainly mediated the observed effects. However, other RSKs may be also important dependent on the cell type because KDM4B can accumulate at DSB sites even in MCF10A cells that do not express RSK4.

DSBs are repaired mainly by two major mechanisms, namely, homologous recombination (HR) and non-homologous end-joining (NHEJ). Studies have differing views on the involvement of KDM4B in DSB repair. Mallette et al. reported that the Tudor domains of KDM4A and KDM4B compete with the Tudor domain of 53BP1, an essential mediator of NHEJ, against H4K20me2 at DSB sites. IR-induced degradation of KDM4A and KDM4B allowed 53BP1 accumulation at DSB sites and induced NHEJ [34]. However, this is inconsistent with KDM4B accumulation at DSB sites in response to DNA damage, as shown in the present and previous reports [9]. In osteosarcoma U2OS cells, the demethylase activity of KDM4B is critical for DSB repair [9]. In drosophila, KDM4A is required for NHEJ, specifically in heterochromatin regions where KDM4A-mediated chromatin relaxation by H3K9 demethylation allows repair factors to access the damaged DNA [35]. Conversely, the inhibition of KDM4A and KDM4B activities by oncometabolites resulted in HR deficiency and impaired accumulation of HR factors including RAD51 at DSB sites by masking the DSB signal with aberrant H3K9me3 hypermethylation and sensitized cells to PARP inhibitors [12, 13]. KDM4B may play an important role in both NHEJ and HR. These complex involvement of KDM4s in DSB repair mechanisms may reflect different roles of KDM4s in hetero- and euchromatins or cell types. The KDM4B-KO cells used in this study did not exhibit impaired accumulation of HR factors or sensitivity to PARPi (Additional File 1: Fig. S6), suggesting that the DSB repair defect in the cells may not be caused by HR deficiency.

KDM4B deletion or the inhibition of its phosphorylation by RSK resulted in IR hypersensitivity. SiRNA depletion of KDM4B was reported to abrogate DSB repair and sensitize osteosarcoma cells to IR [36]. The inhibition of RSK4 by siRNA or BI-D1870 also abrogates DSB repair and sensitizes esophageal cancer cells to IR [37]. The phosphorylation of KDM4B by RSK4 shown in this study could be involved in the previous observation. Conversely, KDM4B inhibition in breast cancer cells induced resistance to TOP2 inhibitors doxorubicin and etoposide [10], whereas the ectopic expression of KDM4B sensitized cells to etoposide [34]. A different mechanism may have been involved in the observations. Seoane et al. reported that the reduced sensitivity to these agents is caused by changes in TOP2 accessibility to DNA due to the condensed state of chromatins [10]. Together with our results, this finding indicates that KDM4B dysfunction affects the sensitivity of cells to DNA-damaging agents differently depending on the source of DNA damage.

Recently, therapeutic targeting of RSKs has been attracting attention [38]. Although BI-D1870 or LJI308 has not been used in the clinic, another RSK inhibitor, PMD-026, is currently in clinical trial for metastatic breast cancer (NCT04115306). The present study uncovered a novel function of RSK on the DNA damage response and provides an additional role of its inhibitor in cancer therapy.

## Conclusions

KDM4B phosphorylation by RSK is critical for KDM4B retention in chromatin for the repair of DNA double-strand breaks, which may provide a new role for RSK inhibitors in cancer therapy.

## Electronic supplementary material

Below is the link to the electronic supplementary material.

Supplementary Material 1

Supplementary Material 2

## Data Availability

No datasets were generated or analysed during the current study.

## Abbreviations

AF-1: Activation function-1
BrdU: Bromodeoxyuridine
CHX: Cycloheximide
DSB: DNA double-strand break
ER: Estrogen receptor
HR: Homologous recombination
IR: Ionizing radiation
KDM4B: Histone lysine demethylase
KO: Knockout
NHEJ: Nonhomologous end-joining
RIPA: Radioimmunoprecipitation assay
RSK: Ribosomal S6 kinase
SERM: Selective estrogen receptor modulators
St2: Tandem-Strep-tag
