# Supplementary material for: Ribosomal S6 kinase (RSK) plays a critical role in DNA damage response via the phosphorylation of histone lysine demethylase KDM4B
Source: Breast Cancer Res. 2024 Oct 21;26:146. doi: 10.1186/s13058-024-01901-x (PMC11492477; doi:10.1186/s13058-024-01901-x)
Supplement: Supplementary file 1 — Supplementary Material 1 [file 13058_2024_1901_MOESM1_ESM.pdf]

Additional file 1

Ribosomal S6 kinase (RSK) plays a critical role in DNA damage response via phosphorylation of lysine demethylase KDM4B

Wenwen Wu, Jing Zhu, Naoe Taira Nihira, Yukiko Togashi, Atsushi Goda, Junki Koike, Kiyoshi Yamaguchi, Yoichi Furukawa, Takuya Tomita, Yasushi Saeki, Yoshikazu Johmura, Makoto Nakanishi, Yasuo Miyoshi and Tomohiko Ohta

Additional file 1 includes 6 figures and 3 tables.

SUPPLEMENTAL FIGURES

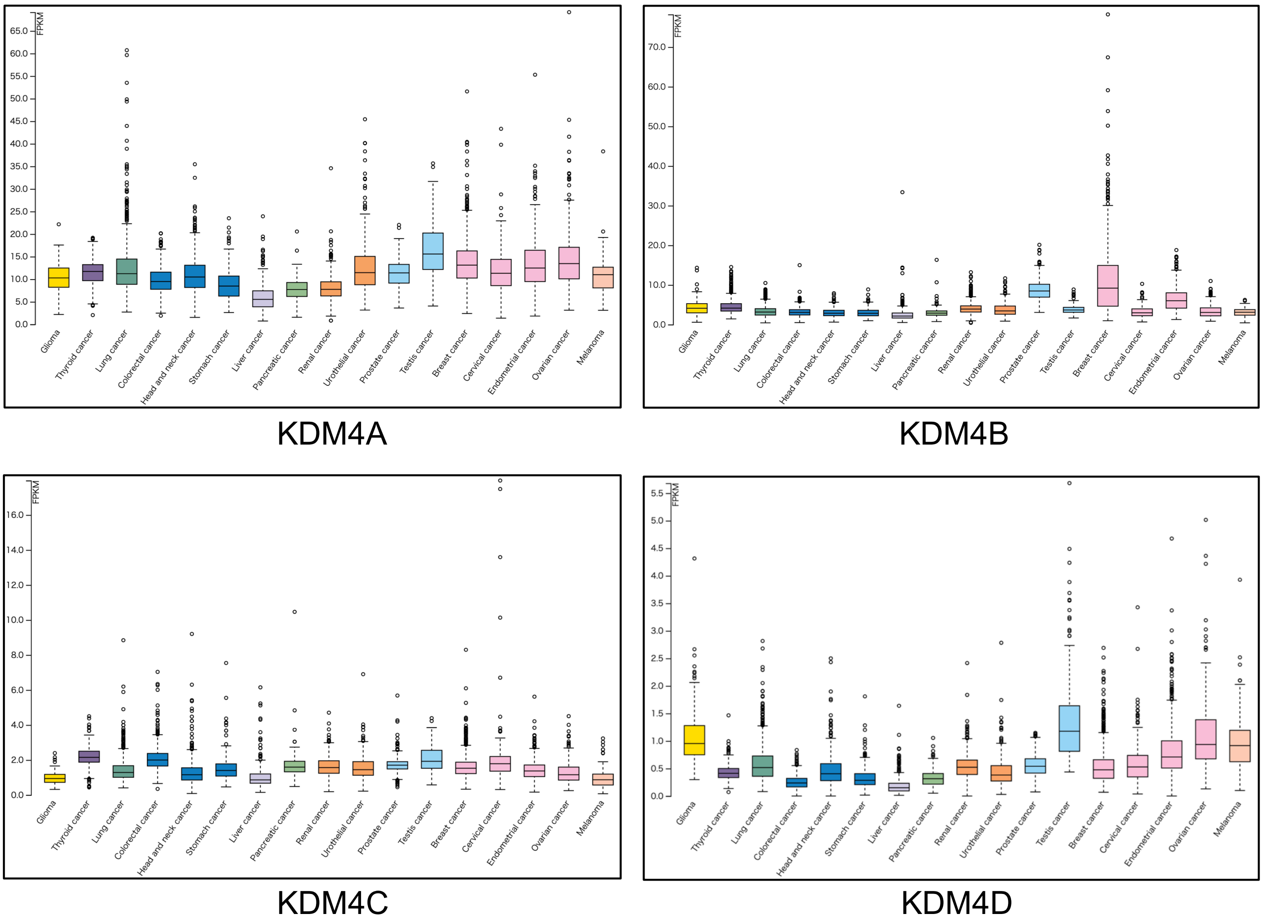

Figure S1. Expression of KDM4 members in various type of cancers from the TCGA dataset. KDM4B expression is significantly higher in breast cancer than in other cancers, whereas no such tissue specificity is found in other family members. KDM4C and KDM4D expression is relatively low in all cancers (note the FPKM scale). FPKM: Fragments Per Kilobase of exon per Million mapped reads. KDM4A [https://www.proteinatlas.org/ENSG00000066135-KDM4A/pathology], KDM4B [https://www.proteinatlas.org/ENSG00000127663-KDM4B/pathology], KDM4C [https://www.proteinatlas.org/ENSG00000107077-KDM4C/pathology], KDM4D [https://www.proteinatlas.org/ENSG00000186280-KDM4D/pathology].

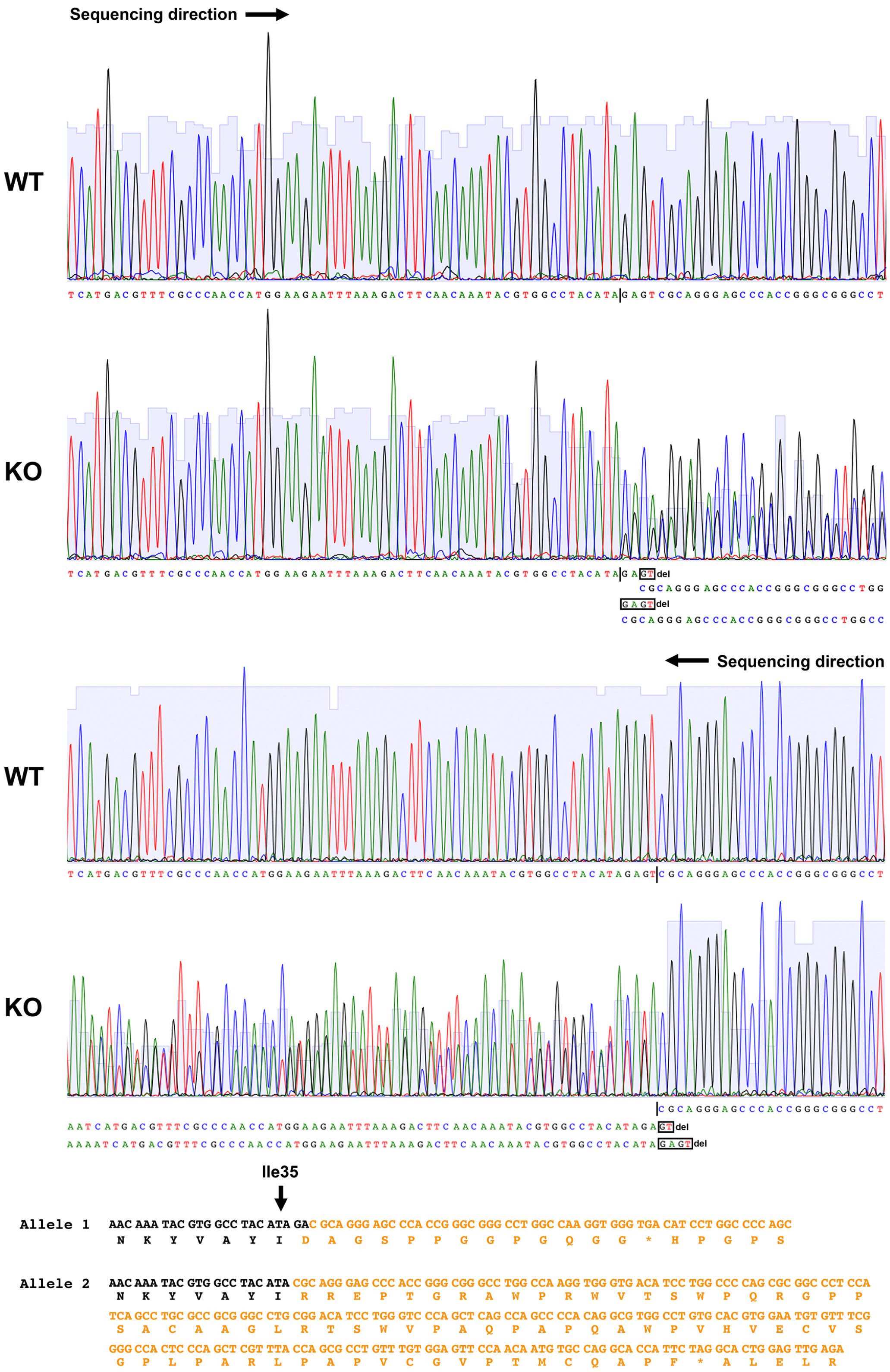

Figure S2. CRISPR/Cas9-mediated knockout of KDM4B in MCF-7 cells.

The sequencing chromatogram of cDNA shows the region with deletion (del) in each allele that causes frameshift after Ile35 (orange letters) and the premature stop codon in KDM4B-KO cells. The deletions have also been verified in genomic DNA.

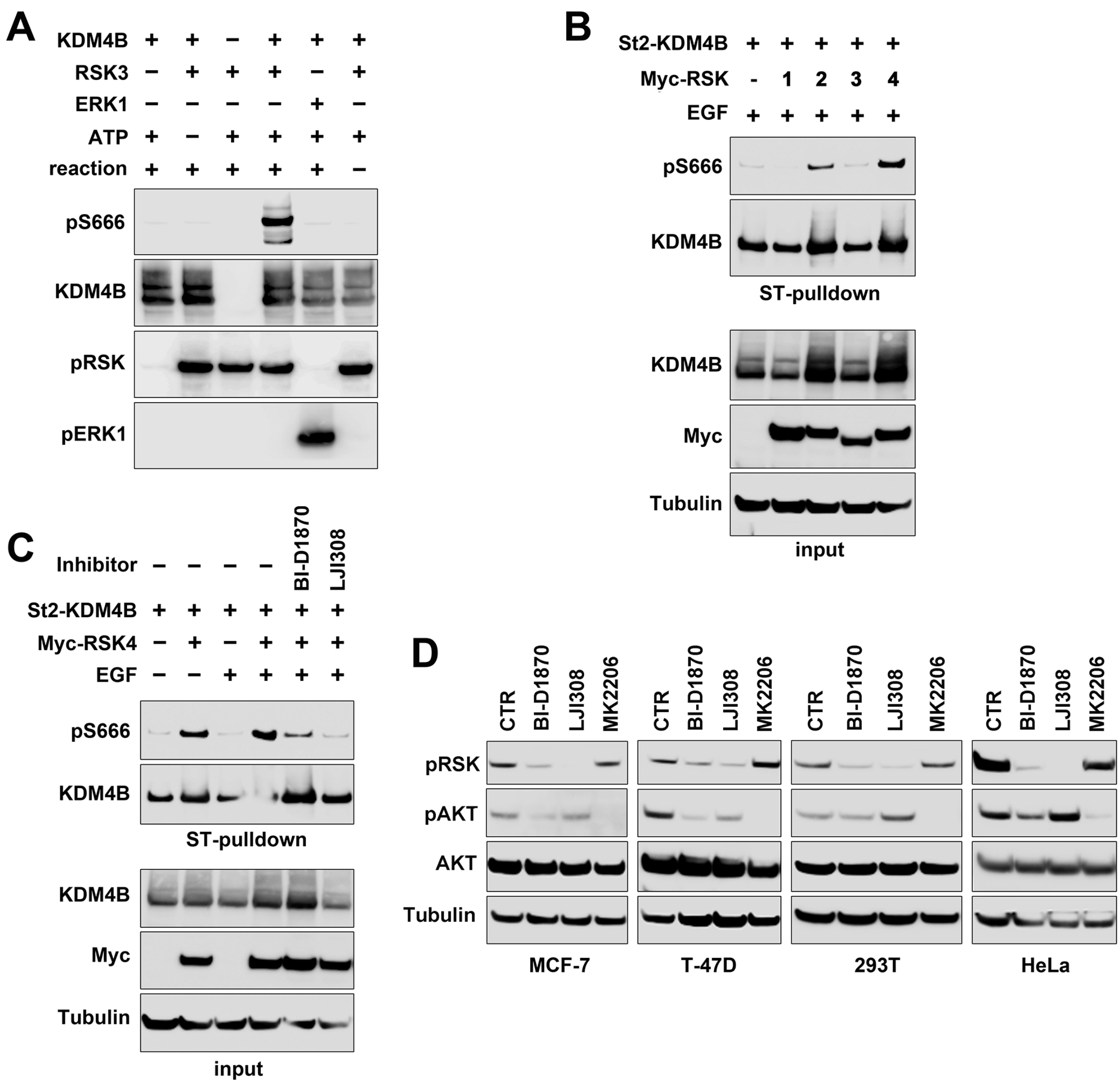

Figure S3. Effect of RSKs and its inhibitors on KDM4B phosphorylation.

(A) Specificity of *in vitro* kinase assay showing KDM4B phosphorylation at Ser666 by RSK3. The indicated recombinant proteins are incubated or not for 10 minutes at 30°C in the presence or absence of ATP and subjected to immunoblotting with the indicated antibodies. KDM4B phosphorylation at Ser666 (pS666) was detected to be dependent on the substrate KDM4B, ATP, reaction, and RSK3, but not ERK1. (B) HEK-293T cells were transfected with St2-KDM4B and Myc-tagged different RSKs, incubated in the presence of EGF, and subjected to StrepTactin (ST) pulldown followed by immunoblotting with the indicated antibodies. Inputs were also loaded. (C) HEK-293T cells were transfected with St2-KDM4B and Myc-RSK4, incubated in the presence or absence of EGF and RSK inhibitors as indicated and subjected to ST pulldown followed by immunoblotting with the indicated antibodies. Inputs were also loaded. (D) MCF-7, T-47D, 293T, and HeLa cells were incubated with the indicated RSK or AKT kinase inhibitors and subjected to immunoblotting with the indicated antibodies.

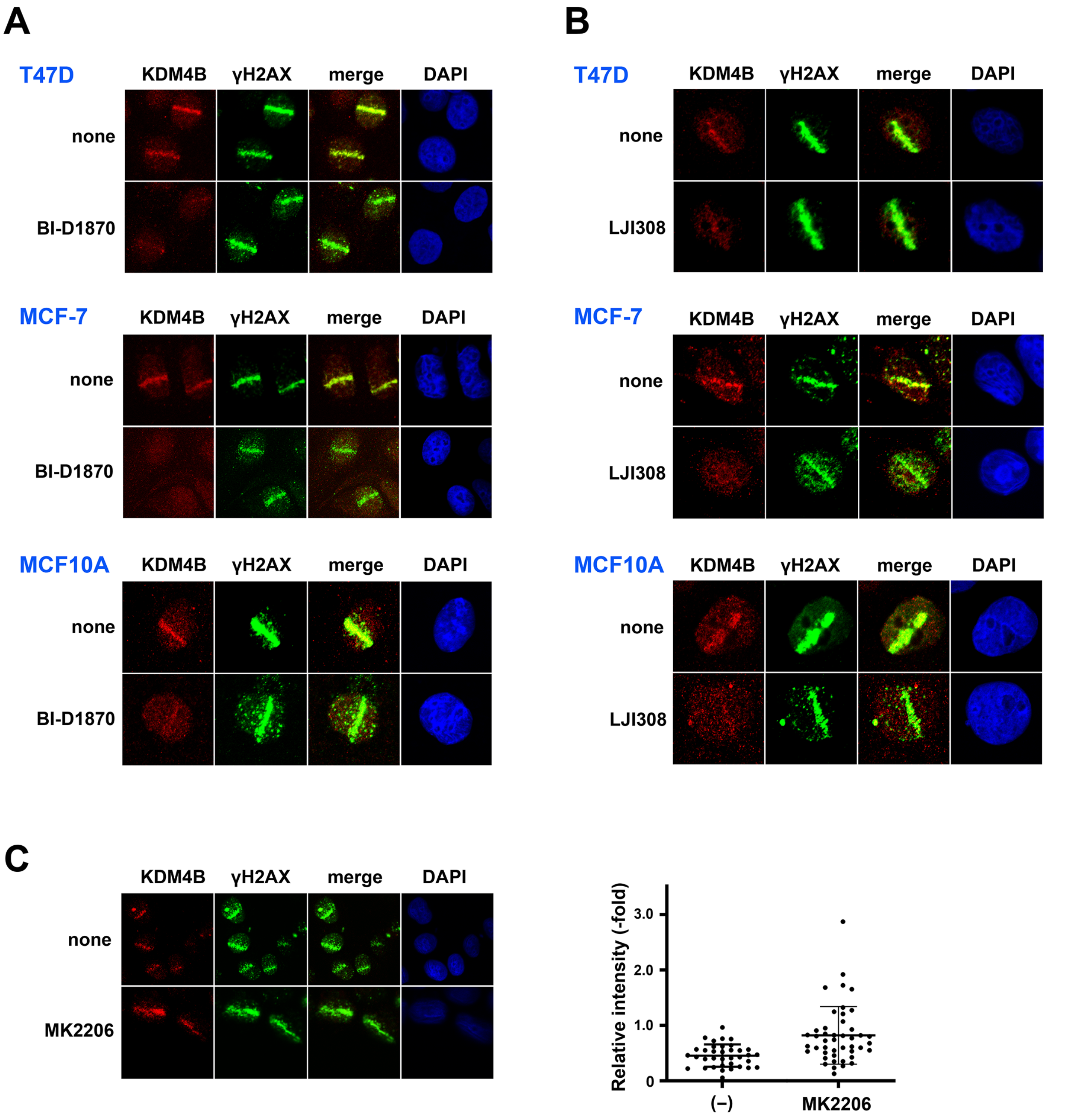

Figure S4. RSK inhibitors, but not AKT inhibitor suppress KDM4B accumulation at DSB sites.

(A and B) Representative data for Fig. 4A and 4B. The indicated cells were incubated with or without BI-D1870 (A) or LJI308 (B), laser microirradiated, and subjected to immunofluorescence. (C) T-47D cells were incubated with or without the AKT inhibitor MK2206 for 24 hours and laser microirradiated followed by immunofluorescence as in Fig. 1A. The relative intensities of KDM4B are shown with means and SDs in the right panels.

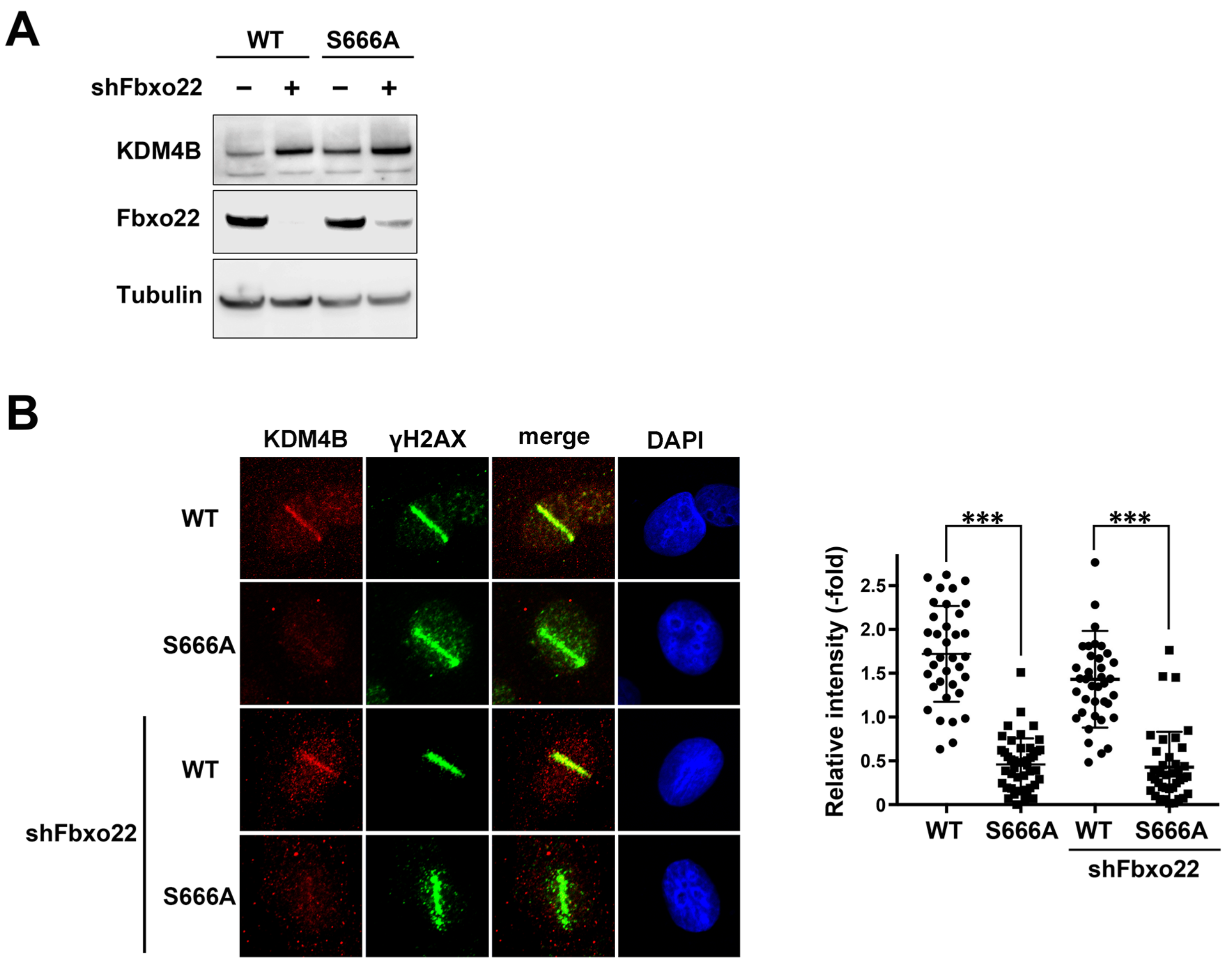

Figure S5. Stabilization by Fbxo22-deletion does not rescue retention of KDM4B-S666A at DSB sites.

(A) WT or KDM4B-S666A MCF-7 cells with or without Dox-inducible shFbxo22 were induced by Dox and subjected to immunoblotting with the indicated antibodies. (B) Cells from (A) were subjected to laser microirradiation followed by immunofluorescence, as in Fig. 1A. The relative intensities of KDM4B are shown as dot plots with means and SDs in the right panel. ***P <0.0001.

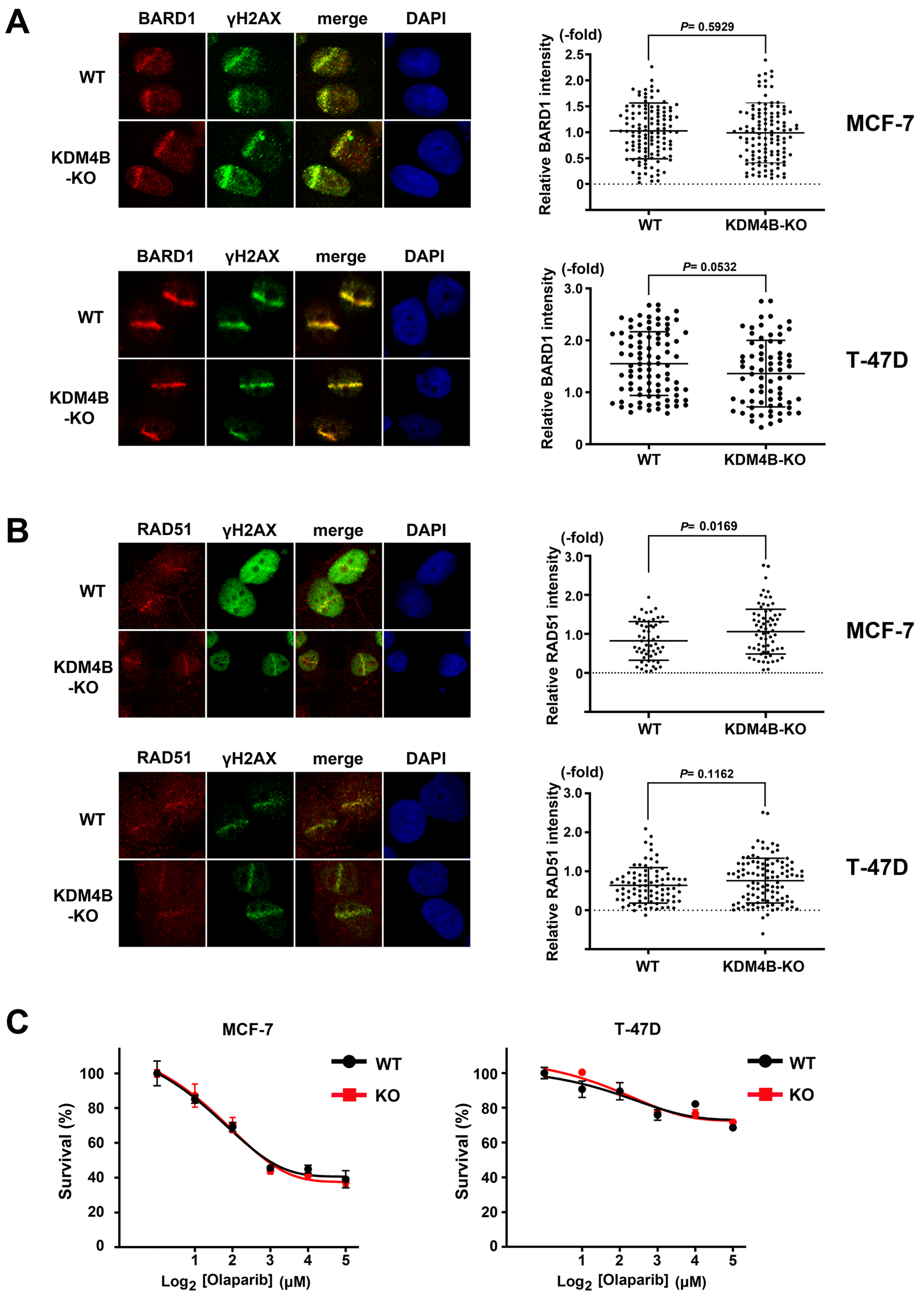
A
BARD1
γH2AX
merge
DAPI
WT
KDM4B
-KO
(-fold)
P= 0.5929
Relative BARD1 intensity
2.5
2.0
1.5
1.0
0.5
0
WT
KDM4B-KO
MCF-7
P= 0.0532
3.0
T-47D
B
RAD51
P= 0.0169
Relative RAD51 intensity
P= 0.1162
C
MCF-7
T-47D
Survival (%)
100
80
60
40
20
KO
Log2 [Olaparib] (μM)

Figure S6. KDM4-KO cells did not show homologous recombination deficiency.

(A and B) WT or KDM4B-KO MCF-7 and T-47D cells were laser microirradiated and then subjected to immunofluorescence as in Fig. 1A with anti-BARD1 (A) or anti-RAD51 (B) antibodies. The relative intensities of KDM4B are shown with means and SDs in the right panels. (C) WT or KDM4B-KO MCF-7 and T-47D cells were treated with olaparib and analyzed for clonogenic survival after 3 weeks. Relative survivals from triplicate experiments are shown as averages ± SDs.

SUPPLIMENTAL TABLES

Table S1. Chemical agents used in this study

Chemicals	Function	Concentration used	Source
BI-D1870	pan-RSK inhibitor	10 µM	Abcam, Cambridge UK
LJI308	pan-RSK inhibitor	10 µM	Selleckchem, Huston TX
MK2206	AKT inhibitor	10 µM	Cayman Chmical, Ann Arbon MI
EGF	growth factor	100 ng/ml	Sigma-Aldrich, St.Louis MO
Olaparib	PARP inhibitor	32 µM	JS Research Chemicals Trading, Wedel DEU

Table S2. antibodies used in this study

Antibodies	Species	Clonality	Source
KDM4B (D7E6)	rabbit	monoclonal	Cell Signaling Tech, Danvers MA
AKT (C67E7)	rabbit	monoclonal	Cell Signaling Tech, Danvers MA
BARD1	rabbit	polyclonal	Bethyl Lab, Montgomery TX
RAD51	rabbit	polyclonal	Bio academia, Osaka Japan
phospho-Ser473-AKT	rabbit	polyclonal	Cell Signaling Tech, Danvers MA
Pan phospho*-RSK	rabbit	polyclonal	Invitrogen, Carlsbad CA
Histone H3	rabbit	polyclonal	Cell Signaling Tech, Danvers MA
Fbxo22	rabbit	polyclonal	GeneTex, Irvine CA
γH2AX (JBW301)	mouse	monoclonal	Millipore, Burlington MA
RSK1 (A-10)	mouse	monoclonal	Santa Cruz Biotech, Santa Cruz CA
RSK2 (E-1)	mouse	monoclonal	Santa Cruz Biotech, Santa Cruz CA
α-tubulin (DM1A)	mouse	monoclonal	Novus Biological, Centennial CO

* phospho-Ser221, Ser227, Ser218, Ser232 of RSK1, 2, 3, 4, respectively

Table S3. Primers used in this study

Target gene	Sequence
RSK1	Forward: CTCATGGAGCTAGTGCCTCTG
	Reverse: GGGATGGATCAGCCTTCTCAG
RSK2	Forward: GCAGAAGATGGCTGTGGAGAG
	Reverse: GTTCAAACTGGGAAGGATCTGCC
RSK3	Forward: GTTCTTCTCTGTGTACCTGCGCAG
	Reverse: GTCCTAAAACCTTCAGCAGCTC
RSK4	Forward: GGTTGATGAGCCAATGGAAGAG
	Reverse: CAGGACCGGTCTTCTTTCTAAC
Actin	Forward: GACCTCTATGCCAACACAGT
	Reverse: AGTACTTGCGCTCAGGAGGA
