# Supplementary material for: Ribosomal S6 kinase (RSK) plays a critical role in DNA damage response via the phosphorylation of histone lysine demethylase KDM4B
Source: Breast Cancer Res. 2024 Oct 21;26:146. doi: 10.1186/s13058-024-01901-x (PMC11492477; doi:10.1186/s13058-024-01901-x)

Additional file 2

Ribosomal S6 kinase (RSK) plays a critical role in DNA damage response via phosphorylation of lysine demethylase KDM4B

Wenwen Wu, Jing Zhu, Naoe Taira Nihira, Yukiko Togashi, Atsushi Goda, Junki Koike, Kiyoshi Yamaguchi, Yoichi Furukawa, Takuya Tomita, Yasushi Saeki, Yoshikazu Johmura, Makoto Nakanishi, Yasuo Miyoshi and Tomohiko Ohta

Additional file 2 includes uncropped images of gels and blots used in this study.

Fig. 1C

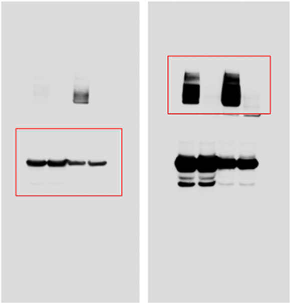

Fig. 2A

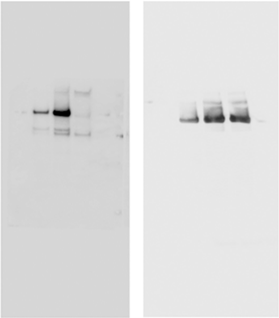

Fig. 2B

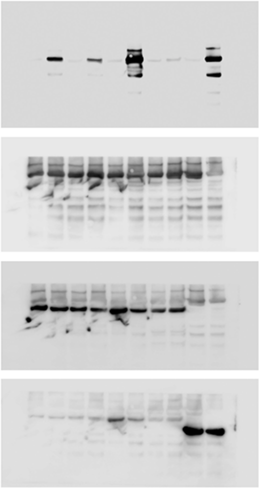

Fig. 2C

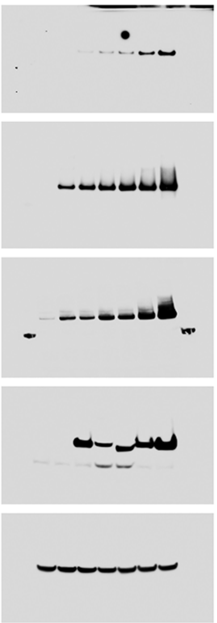

Fig. 2D

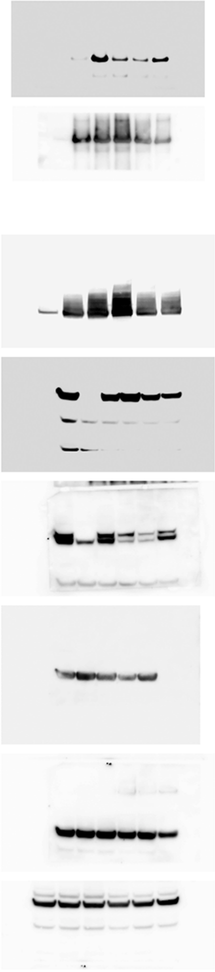

Fig. 2E

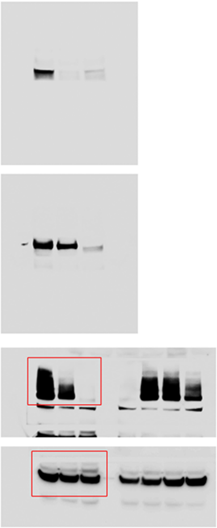

Fig. 2F

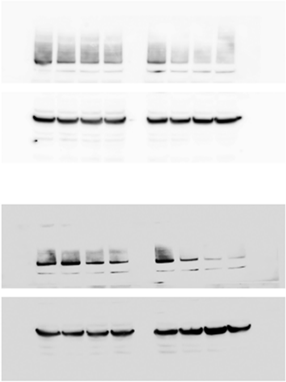

Fig. 3A

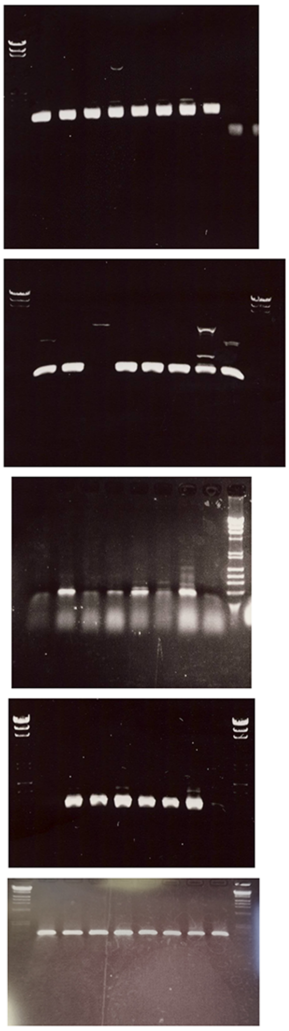

Fig. 3B

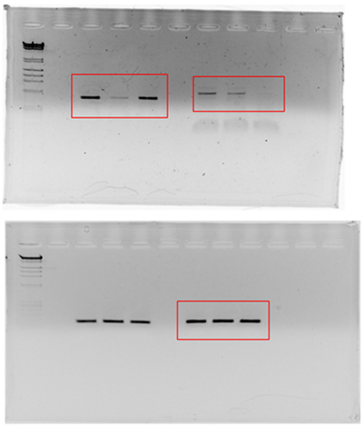

Fig. 3D

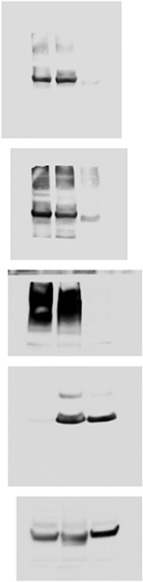

Fig. 3E

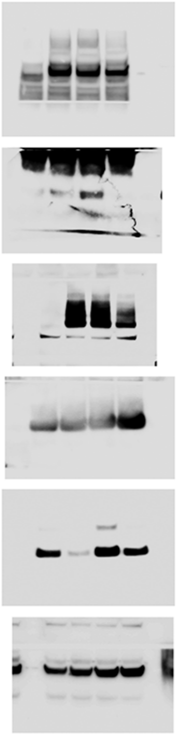

Fig. 5A

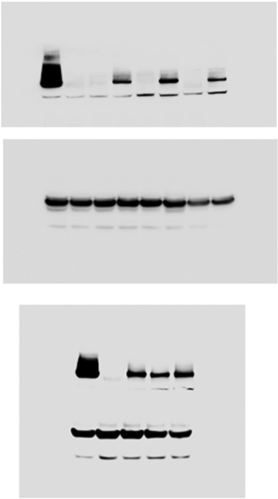

Fig. S3A

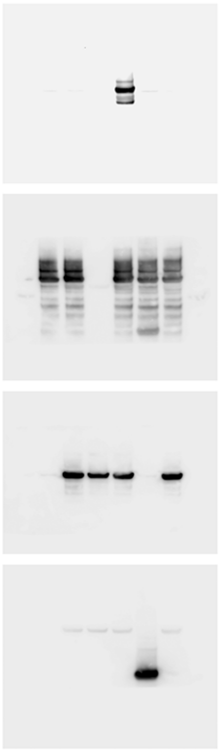

Fig. S3B

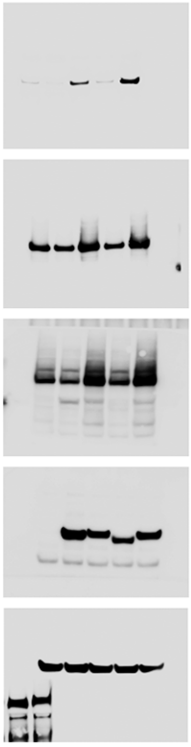

Fig. S3C

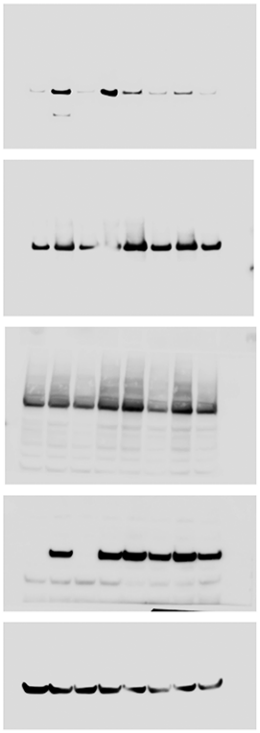

Fig. S5A

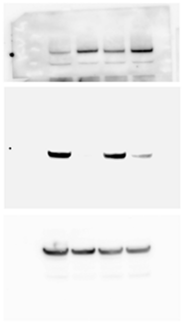

Fig. S3D

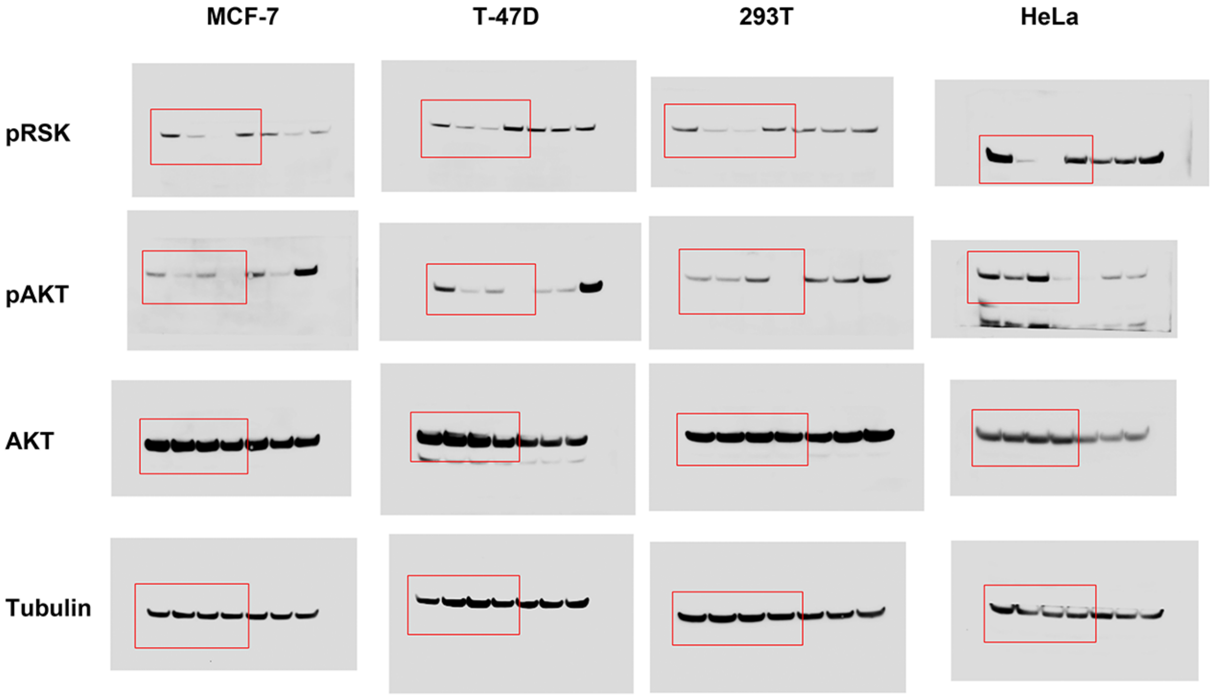

Supplement: Supplementary file 2 — Supplementary Material 2 [file 13058_2024_1901_MOESM2_ESM.pdf]
